# Arterial reconstruction using the donor’s gonadal vein in living renal transplantation with multiple renal arteries: a case report and a literature review

**DOI:** 10.1186/s12882-020-01848-z

**Published:** 2020-05-20

**Authors:** Mitsuru Tomizawa, Shunta Hori, Nobutaka Nishimura, Chihiro Omori, Yasushi Nakai, Makito Miyake, Tatsuo Yoneda, Kiyohide Fujimoto

**Affiliations:** Department of Urology, Nara Medical University, 840 Shijo-cho, Kashihara, Nara 634-8522 Japan

**Keywords:** Kidney transplantation, Gonadal vein, Arterial reconstruction, Multiple arteries, Case report

## Abstract

**Background:**

Arterial reconstruction is one of the paramount procedures in kidney transplantation (KT) and greatly important if the procured kidney has multiple renal arteries (MRA). Despite various established techniques for arterial reconstruction, sometimes, the surgeon finds performing arterial anastomoses challenging in case of MRA. In our case, the donor’s gonadal vein and recipient’s internal iliac artery graft were used for arterial anastomoses, and 3 years after KT, the allograft did not present vascular complications.

**Case presentation:**

A 34-year-old man underwent ABO-incompatible preemptive living KT. The allograft had three renal arteries and four renal veins. After donor nephrectomy, arterial reconstruction was performed on a back table. These arteries were reconstructed into one piece using the recipient’s internal iliac artery graft. The two arteries at the middle of the renal hilum were reconstructed using the conjoined method. As the superior renal artery was too short to anastomose, the donor’s gonadal vein was used for extension. The internal iliac artery graft was anastomosed to the original internal iliac artery. Intraoperative Doppler ultrasonography revealed that the blood flow in each renal artery was adequate, resulting in sufficient blood flow throughout the allograft. The allograft function was maintained with a serum creatinine level of approximately 0.9 mg/dL without vascular complications 3 years after KT.

**Conclusions:**

The donor’s gonadal vein can be a candidate for extension of the renal artery in the allograft with MRA. Further follow-up is needed for the assessment of long-term outcomes.

## Background

Kidney transplantation (KT) is the most effective treatment for end-stage renal disease. Living donor allografts are widely used because of the persistent shortage of cadaveric kidneys, and allografts with multiple renal arteries (MRA) are sometimes used. Autopsy studies have shown that 17% of kidneys have MRA [1]. Another study has shown that 12.8% of allografts had MRA in living KT [2]. The use of allograft with MRA associated with a lower 1-year graft survival, a higher complication rate and an increase frequency of delayed graft function compared to the use of allograft with single renal artery [3]. However, MRA allografts had no effect on the 5-year graft survival and 1- and 5-year patient survival [3]. Although selection of allografts for MRA is controversial, a donor kidney which has better function must be avoided for allograft if there is a difference between the right and left sides. For MRA, arterial reconstruction is of paramount importance. Various techniques for arterial reconstruction have been reported, including the conjoined method, end-to-side method, and the use of the recipient’s internal iliac artery graft. The effectiveness and safety of these methods have also been reported [4]. In cases of more than three arteries, arterial reconstruction can be difficult even with the use of these methods [5]. In our case, arterial reconstruction for four renal arteries was needed. Herein, we report the elongation of the renal artery with the use of the donor’s gonadal vein, which resulted in no evidence of vascular complications 3 years after KT.

## Case presentation

**Fig. 1:**
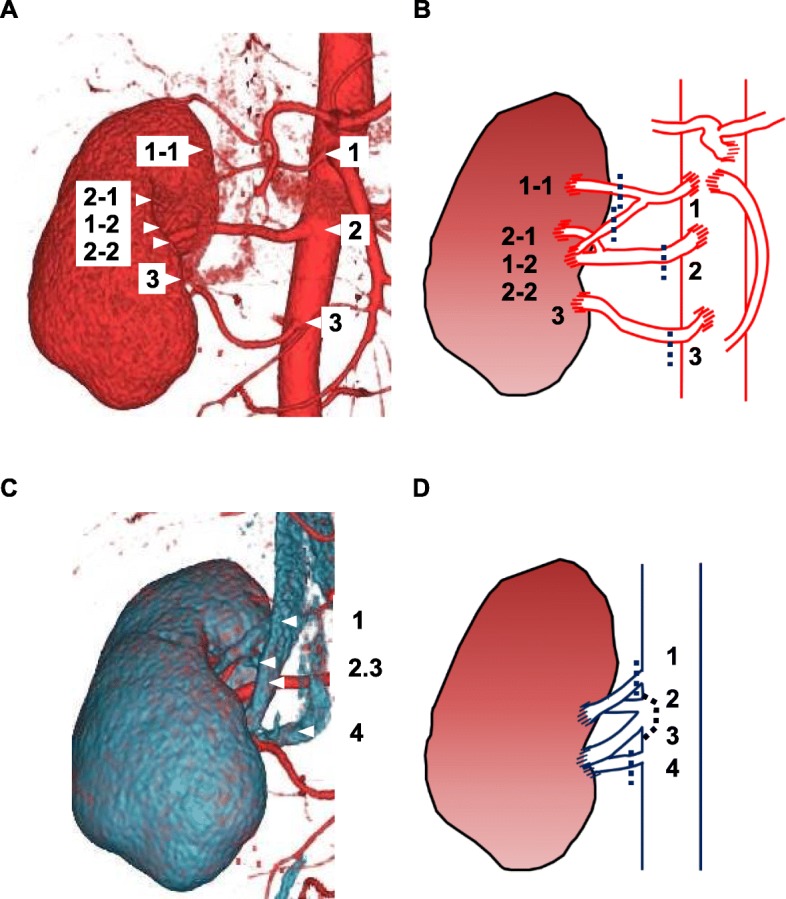
Three-dimensional computed tomography images and schemas of the renal blood vessels. Three renal arteries branched from the aorta, and the superior and middle arteries (1, 2) branched into two arteries (1-1, 1-2, 2-1, 2-2) (**a**). The renal arteries were cut at the dotted line (**b**). The superior artery was cut distal to the branch point due to bleeding. Four renal veins branched from the vena cava (**c**). The two veins at the middle (2, 3) were cut simultaneously with the vena cava wall (**d**)

**Fig. 2:**
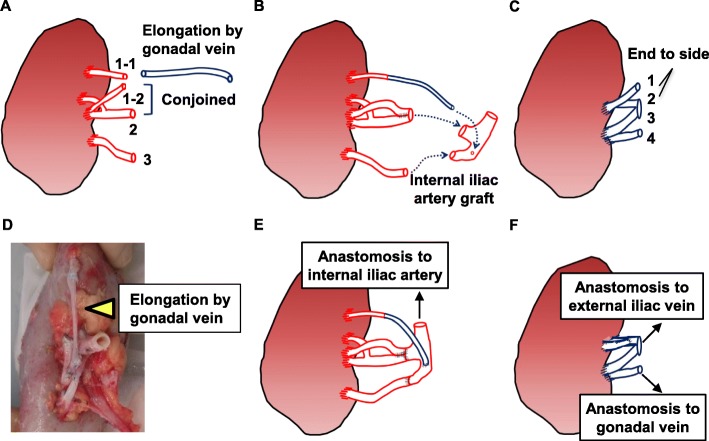
Schema and image of reconstruction of the renal blood vessels. The two arteries at the middle of the renal hilum were reconstructed using the conjoined method, and the superior renal artery was lengthened using the donor’s gonadal vein (**a**). The renal arteries were reconstructed into one using the internal iliac artery graft (**b**). The superior two veins (1, 2) were reconstructed using the end-to-end method (**c**). **d** Representative images of the reconstruction. The yellow arrow shows the gonadal vein graft. The internal iliac artery graft was anastomosed to the original internal iliac artery (**e**), and the renal veins were anastomosed to the external iliac and gonadal veins (**f**)

**Fig. 3:**
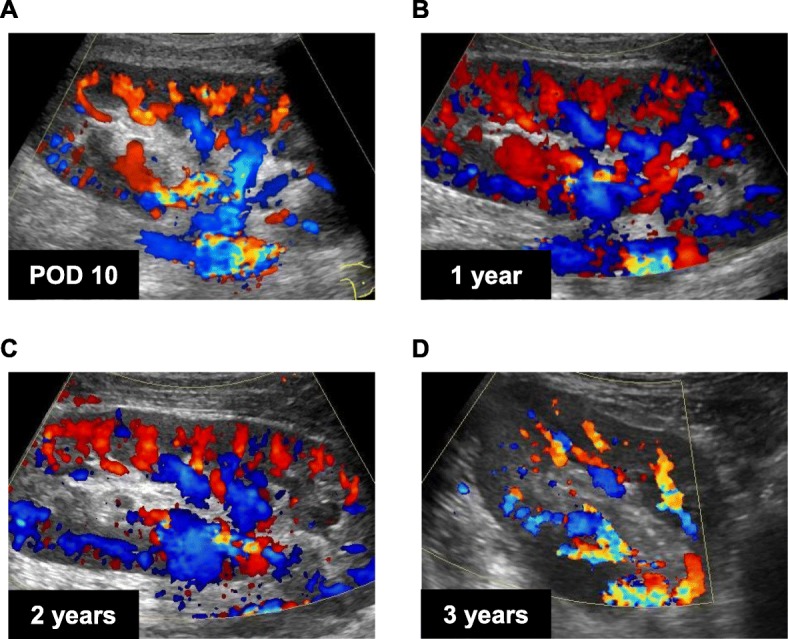
Representative images of the allograft obtained by Doppler ultrasonography (**a**, postoperative day 10; **b**, 1 year post-transplantation; **c**, 2 years post-transplantation; **d**, 3 years post-transplantation)

A 34-year-old man was hospitalized for preemptive living KT. He was diagnosed with end-stage renal disease secondary to IgA nephropathy 1 year prior. He underwent ABO-incompatible living KT. The donor was his brother, and his right kidney was selected for allograft because the 99 m-Tc-diethylenetriamine pentaacetic acid renogram revealed that the glomerular filtration rate of the right kidney was > 10% of that of the left kidney. The allograft had three renal arteries (Fig. 1a, b) and four renal veins (Fig. 1c, d). The renal arteries and veins were cut at the dotted line shown in Fig. 1b and d. The donor’s gonadal vein was harvested for vascular graft because of vascular complexity. The diameter of gonadal vein was approximately 3 mm. The renal arteries and veins were trimmed and prepared on the back table, as shown in Fig. 2a, b, and c. The renal arteries were reconstructed into one piece using the internal iliac artery graft obtained from the recipient by interrupted 7/0 Proline stitches. The two arteries, both had a diameter approximately 5 mm, at the middle of the renal hilum were reconstructed into one piece using the conjoined method by interrupted 7/0 Proline stitches. The superior renal artery was too short to anastomose to the internal iliac artery graft. The inferior epigastric artery was insufficient for anastomosis to the renal artery or interposition. The diameter of inferior epigastric artery was approximately 1 mm, compared to that of superior renal artery was approximately 3 mm. Although an interposition method using an artificial blood vessel graft was also considered, we decided to use the gonadal vein graft obtained from the donor for long-term patency of the graft. The superior renal artery was lengthened using the donor’s gonadal vein and subsequently anastomosed to the internal iliac artery graft (Fig. 2d). The internal iliac graft was anastomosed to the original internal iliac artery (Fig. 2e) using 6/0 Proline suture in an interrupted fashion. The renal veins were anastomosed to the external iliac vein and the recipient’s gonadal vein (Fig. 2f). Intraoperative Doppler ultrasonography (US) revealed that the blood flow in each renal artery was adequate, resulting in sufficient blood flow throughout the allograft. Urine output was observed immediately after the blood flow returned. Doppler US showed no evidence of anastomotic stenosis, obstruction, or aneurysm of the renal arteries on postoperative day (POD) 10 (Fig. 3a), 17, 24. There were no surgical complications, and the patient was discharged on POD 32. The renal arteries and allograft were evaluated annually by Doppler US (Fig. 3b, c, and d). The renal graft function has been maintained with a serum creatinine level of approximately 0.9 mg/dL, and there was no evidence of vascular complications on Doppler US performed 3 years after KT.

## Discussion and conclusion

We provided evidence that extension of the transplant renal artery using the gonadal vein was acceptable in a case with MRA. The course of this patient suggested that the donor’s gonadal vein grafts might have good patency and safety, at least in the short to medium term. To the best of our knowledge, 6 cases treated with the use of the gonadal vein graft (regardless of the donor’s gonadal vein or recipient’s) have been reported, including our case (Table 1) [5–8]. In 5 of these 6 cases, the donor’s gonadal vein was grafted. In these cases, the donor’s gonadal veins were intentionally harvested for the graft. It is easy to harvest the donor’s gonadal vein during donor nephrectomy. In the remaining case, the recipient’s gonadal vein was harvested and used for extension of the renal artery because of shortening of the renal artery due to bleeding during donor nephrectomy. Furthermore, no vascular complications were noted during short-term follow-up in these 6 cases. However, long-term patency and safety remain unclear.

**Table 1:** Summary of previous reports of arterial reconstruction using a gonadal vein

	Authors	Year	Age	Sex	Number of allograft renal arteries	Source of the gonadal vein	Reason for use	Anastomosed to	Follow-up period
1	Hakaim AG	1992	NA	NA	2	donor	multiple renal arteries	external iliac artery	1 year 6 months
2	Hakaim AG	1992	NA	NA	2	donor	multiple renal arteries	inferior epigastric artery	3 weeks
3	Chatzizacharias NA	2010	28	M	2	donor	multiple renal arteries	external iliac artery	5 days
4	He B	2012	56	F	3	donor	multiple renal arteries	external iliac artery	2 months
5	Uysal E	2017	27	M	1	recipient	inadequate artelial length	internal iliac artery	8 months
6	Present case	2020	34	M	3	donor	multiple renal arteries	internal iliac artery graft	3 years

*Pt* patient, *NA* not available

The saphenous vein is most widely used in venous bypass graft procedures, while the gonadal vein is rarely used. Generally, saphenous vein grafts are commonly placed in the coronary and lower extremity vasculature. In the coronary artery, saphenous vein grafts and radial artery grafts are used for coronary-artery bypass grafting. A randomized controlled trial revealed that, compared with the use of radial artery grafts, the use of saphenous vein grafts was associated with a higher risk of occlusion [9]. The occlusion rates were 19.9% in the saphenous vein graft group and 8.1% in the radial artery graft group at follow-up angiography (mean follow-up, 50 ± 30 months). The etiology of failure is thrombosis within the first month, intimal hyperplasia from 1 to 12 months, and atherosclerotic degeneration or progression of underlying arterial disease after 12 months [10]. In the lower extremity, saphenous vein and artificial blood vessel grafts (polytetrafluoroethylene) are used for femoropopliteal bypass grafting. Compared with the use of artificial blood vessel grafts, the use of saphenous vein grafts was associated with a lower risk of occlusion [11]. The 5-year graft patency rate was 68.9–77.2% in the saphenous graft group but 48.3–57.4% in the artificial blood vessel graft group [11]. Lower extremity grafts have a higher rate of early graft failure due to intimal hyperplasia and valve sclerosis and late failure due to progression of native vasculature. Considering these studies, the patency rate of artery grafts seems excellent, and the patency rate of vein grafts seems better than that of artificial blood vessel grafts, but it is unclear whether the etiology of graft failure in these cases can be extrapolated to the etiology of graft failure in KT.

Generally, antiplatelet therapy is used to prevent saphenous vein graft occlusion [12, 13]. In KT, there is no evidence of vein graft occlusion because vein grafts are rarely used for artery bypass grafting. The use of postoperative thromboprophylaxis for graft thrombosis is also controversial in KT [14]. Although postoperative thromboprophylaxis was not used in this case, further studies should be accumulated to evaluate thrombosis risk in vein grafts.

Saphenous vein grafts are also the most common type of renal aortorenal bypass grafts in the treatment of renovascular diseases. Dean et al. reported long-term complications of renal aortorenal bypass grafts in 29 patients with 39 saphenous vein grafts [15]. Two of the 39 vein grafts (5%) developed an aneurysm. The development of aneurysmal change was recognized in these grafts 3 and 6 years after bypass grafting. In other studies, 3 cases of venous graft rupture after renal aortorenal bypass grafting have been reported [16–18], wherein rupture occurred 19 years, 22 years, and 30 years after bypass grafting. These cases suggest that long-term follow-up with Doppler US should be recommended in cases wherein the donor’s gonadal vein grafts are used.

In conclusion, arterial reconstruction using the donor’s gonadal vein is acceptable for an allograft with MRA, although other techniques, such as the conjoined method, end-to-side method, and recipient’s internal iliac artery graft, should be prioritized. Further follow-up using Doppler US is needed for the assessment of long-term outcomes, and further reports should be accumulated to determine long-term patency and safety.

## Data Availability

Records and data pertaining to this case are in the patient’s secure medical records in the Nara Medical University.

## Abbreviations

KT: Kidney transplantation
MRA: Multiple renal arteries
US: Ultrasonography
POD: Postoperative day
